# Meningioma cells express primary cilia but do not transduce ciliary Hedgehog signals

**DOI:** 10.1186/s40478-020-00994-7

**Published:** 2020-07-20

**Authors:** Sarah Findakly, Abrar Choudhury, Vikas Daggubati, Melike Pekmezci, Ursula E. Lang, David R. Raleigh

**Affiliations:** 1 Department of Radiation Oncology, University of California San Francisco, San Francisco, CA USA; 2 Department of Neurological Surgery, University of California San Francisco, San Francisco, CA USA; 3 Department of Pathology, University of California San Francisco, San Francisco, CA USA; 4 Department of Dermatology, University of California San Francisco, San Francisco, CA USA

**Keywords:** Cilia, Hedgehog, Meningioma, Primary cilium, Smoothened, Vismodegib

## Abstract

Meningiomas are the most common primary intracranial tumors, but treatment options for meningioma patients are limited due to incomplete understanding of tumor biology. A small percentage of meningiomas harbor somatic variants in the Hedgehog pathway, a conserved gene expression program that is essential for development and adult stem cell homeostasis. Hedgehog signals are transduced through primary cilia, and misactivation of the Hedgehog pathway is known to underlie cancer. Nevertheless, the mechanisms of Hedgehog signaling in meningioma are unknown. Here, we investigate mechanisms of ciliary Hedgehog signaling in meningioma using tissue microarrays containing 154 human meningioma samples, NanoString transcriptional profiling, primary meningioma cells, pharmacology, and CRISPR interference. Our results reveal that meningiomas of all grades can express primary cilia, but that cilia are less prevalent among anaplastic tumors. Moreover, we find that expression of Smoothened alleles that are oncogenic in other contexts fail to activate the Hedgehog transcriptional program or promote proliferation in primary meningioma cells. These data reveal that meningiomas can express the subcellular structure necessary for canonical Hedgehog signaling, but suggest that they do not transduce ciliary Hedgehog signals.

## Introduction

Meningiomas arising from the lining of the central nervous system are the most common primary intracranial tumors [15]. The World Health Organization (WHO) classifies meningiomas according to mitotic activity and histopathologic features associated with aggressive clinical behavior [13]. WHO grade I meningiomas have low mitotic activity and can be effectively treated with surgery and radiation [3]. In contrast, high grade meningiomas, which account for 20–30% of cases, are a significant cause of neurologic morbidity and mortality [3]. Thus, there is an urgent, unmet need for new meningioma treatments. However, new therapies for meningioma patients have been encumbered by limited understanding of meningioma biology, and a lack of druggable mechanisms underlying meningioma growth [3].

A very small percentage of meningiomas harbor somatic variants in the Hedgehog pathway [2, 4, 5, 22], a conserved gene expression program that is essential for development and adult stem cell homeostasis [9]. Misactivation of the Hedgehog pathway can cause cancer, including basal cell carcinoma, the most common cancer in the United States, and medulloblastoma, the most common malignant brain tumor in children [18]. Meningiomas express Hedgehog genes [12], and the majority of Hedgehog pathway variants in meningiomas are mutually exclusive from *NF2* variants. However, Hedgehog pathway mutations are found in low grade meningiomas with favorable outcomes after treatment with standard therapies [19]. Moreover, over-expression of an oncogenic allele of the Hedgehog pathway activator Smoothened (SMO) promotes arachnoid hyperproliferation in mice, but does not cause meningiomas of the same size or number as inactivating alleles of *Nf2* [1]. These data suggest that the mechanisms of Hedgehog signaling in meningiomas remain incompletely understood.

Vertebrate Hedgehog signals are transduced through the primary cilium, an antennae that projects from the surface of most cells [7]. Hedgehog ligand binding to Patched (PTCH) allows SMO to accumulate in cilia and activate the GLI family of transcription factors, which translocate to the nucleus and activate the Hedgehog transcriptional program [9]. Primary cilia are necessary for canonical Hedgehog signal transduction, but are not sufficient to activate the Hedgehog transcriptional program, which is controlled by mechanisms downstream of cilia. A clinical trial exploring the efficacy of the SMO inhibitor vismodegib in meningioma was initiated (NCT02523014), but vismodegib inhibits ciliary SMO, and it is unknown if meningiomas express primary cilia or encode the Hedgehog transcriptional program.

## Methods

### Cell culture and small molecule treatments

The primary human meningioma M10G cell line was derived from a fresh meningioma resection (WHO grade I, *NF2* wild type, Chr22q loss of 1 copy, convexity) by mechanically mincing approximately 100 mg of tumor tissue in Hanks’ Balanced Salt Solution (HBSS) and then plating in media with a 1:1 ratio of Dulbecco’s Modified Eagle Medium (DMEM) and F12 (Life Technologies, #10565) and Neurobasal medium (Life Technologies, #21103), supplemented with 5% fetal bovine serum (FBS) (Life Technologies, #16141), B-27 supplement without vitamin A (Life Technologies, #12587), N-2 supplement (Life Technologies, #17502), 1X GlutaMAX (Life Technologies, #35050), 1 mM NEAA (Life Technologies, #11140), 100 U/mL Anti-Anti (Life Technologies, #15240), 20 ng/mL EGF (R&D systems, Minneapolis, MN, #236-EG), and 20 ng/mL FGF2 (Peprotech, Rocky Hill, NJ, #100-18C).

The primary human meningioma BenMen cell line (WHO grade I, *NF2*-mutant, falx) was originally derived from a meningothelial meningioma and transfected with hTERT to achieve immortalization [17]. Cells were cultured in DMEM (10313021, Life Technologies) supplemented with 10% FBS and 1X GlutaMAX, which was also used to culture NIH3T3 (CRL-1658, ATCC) and HEK293T (CRL-3216, ATCC) cells.

For ciliation and Hedgehog signaling assays, NIH3T3, BenMen and M10G cultures were transitioned to OptiMEM (31985062, Thermo Fisher Scientific) and treated with recombinant Sonic Hedgehog 1 μg/ml (1845, R&D Systems), Smoothened agonist 100 nM (566660, Calbiochem) or vehicle control, for 24 h.

### Cell transfection

*SMO*, the oncogenic allele *SMO*^*W535L*^, and the somatic variant *SMO*^*L412F*^ were cloned into the pEGFP vector, and constitutively active GLI2 (GLI2^CLEG^) was cloned into pCMV vector. According to the manufacturer’s instructions, Fugene HD reagent (Promega, E2311) was used for transfection of constructs into NIH3T3, BenMen and M10G cells. Cells were harvested for experimentation 72 h after transfection.

### CRISPR interference

Lentiviral particles containing pMH0001 (UCOE-SFFV-dCas9-BFP-KRAB, Addgene #85969) were produced by transfecting HEK293T cells with standard packaging vectors using the *Trans*IT-Lenti Transfection Reagent (Mirus Bio, MIR 6605). M10G cells were stably transduced with lentiviral particles to generate M10G^dCas9-KRAB^ cells by isolating BFP-expressing cells using fluorescence activated cell sorting on a Sony SH800.

Single-guide RNA (sgRNA) protospacer sequences were individually cloned into the pCRISPRia-v2 vector (Addgene plasmid #84832), between the BstXI and BlpI sites, by ligation. Each vector was verified by Sanger sequencing of the protospacer. One sgRNA expression vector with the following protospacer was cloned for non-targeting control sgRNAs (ncRNA) (5′-GCTGCATGGGGCGCGAATCA-3′), *PTCH1* sgRNAs (sg*PTCH1*) (5′-AAATGTACGAGCACTTCAAG-3′) and *SMO* sgRNAs (sg*SMO*) (5′-CAAGAACTACCGATACCGTG-3′). Lentivirus was generated as described above for each sgRNA expression vector, and M10G^dCas9-KRAB^ cells were independently transduced with lentivirus from each sgRNA expression vector, then selected to purity using 20 μg/mL puromycin over 7 days.

### Meningiomas

Meningioma tissue microarrays were comprised of cases treated with surgery at the University of California San Francisco (UCSF) between 2003 to 2012. Demographic and clinical data were obtained from the medical record. For all cases, diagnostic imaging was re-reviewed to confirm the extent of resection and determine the occurrence and timing of local recurrence, which was defined as local recurrence of any size after gross-total resection, or growth of ≥20% along any dimension after subtotal resection. Mortality data and cause of death were extracted from the electronic medical record, institutional cancer registry, Surveillance, Epidemiology, and End Results (SEER), Department of Motor Vehicles (DMV), Social Security, and nationwide hospital databases, and publicly available obituaries. Overall survival was defined as the length of time from resection of meningioma to death. This study was approved by the Institutional Review Board, Human Research Protection Program Committee on Human Research, protocol 18–24,633.

### Meningioma and meningioma tissue microarray ciliary immunofluorescence

Meningioma tissue microarrays were created as previously described [14]. In brief, two 2 mm cores were taken from each meningioma block, along with control tissue from normal adult brain, normal adult meninges, 2 lung adenocarcinomas, and placenta. All meningioma diagnoses and grades were re-reviewed according to current diagnostic criteria by a board-certified neuropathologist (MP) [13]. Only patients with demographic, histopathologic, radiologic, and comprehensive clinical follow-up data who consented to tumor sampling for research were included. Ciliary immunofluorescence was performed as previously described [11]. In brief, sections were deparaffinized in xylene, rehydrated through graded ethanol dilutions and subjected to antigen retrieval using CC1 TRIS buffer (Ventana Medical Systems); labeled with primary antibodies including Pericentrin (PA5–54109, Thermo Fisher Scientific) and γTubulin (T5192, Sigma) to mark centrosomes, and acetylated tubulin to mark cilia (T6793, Sigma). Alexa Fluor secondary antibodies and DAPI (62,248, Thermo Fisher Scientific) were used, and sections were mounted in mounted in ProLong Diamond Antifade Mountant (Thermo Fisher Scientific).

### Microscopy

Fluorescence microscopy was performed on a Zeiss LSM 800 confocal laser scanning microscope with a PlanApo 20X air objective. Images were processed and quantified from a representative region of each tumor using ImageJ. Cilia prevalence was quantified as the ratio of cilia to nuclei. Cilia length was quantified using a region of interest calculation.

### NanoString transcriptional profiling

NanoString transcriptional profiling from formal-fixed paraffin-embedded meningiomas was performed as previously described [21]. In brief, total RNA was extracted from tumor cores containing 75% or more tumor cells, as determined by hematoxylin and eosin (H&E) staining. The GX Human Cancer Reference NanoString panel codeset, containing 30 additional meningioma related genes, were synthesized by NanoString Technologies. Of note, *SMO* was the only Hedgehog pathway gene included in this panel. RNA (200 ng per meningioma) was analyzed by the NanoString nCounter Analysis System at NanoString Technologies, according to the manufacturer’s protocol. Data were preprocessed, normalized against a panel of housekeeping genes, and log2-transformed count data were centered and scaled within-meningiomas using a Z-score transformation.

### Primary meningioma cell immunofluorescence

Immunofluorescence for acetylated tubulin (AcTub) and Ki67 from meningioma cells was performed on glass coverslips. Cells were fixed in 4% paraformaldehyde, blocked in 2.5% BSA (Sigma) and 0.1% Triton X-100 (Sigma) in Phosphate Buffered Saline (PBS) for 30 min at room temperature (Thermo Fisher Scientific), and labeled with anti-Ki67 (ab15580, Abcam), anti-Smoothened (20787–1-AP, ProteinTech), anti-Centriolin (sc-365,521, Santa Cruz), anti-Arl13b (17711–1-AP, ProteinTech) or anti-acetylated tubulin (T6793, Sigma) primary antibodies at room temperature for 3 h. Cells were labeled with Alexa Fluor secondary antibodies and DAPI to mark DNA (Life Technologies, H3570) for 1 h at room temperature, and were mounted in ProLong Diamond Antifade Mountant (Thermo Fisher Scientific).

### Quantitative reverse transcriptase polymerase chain reaction

RNA was isolated from cells using the RNEasy Mini Kit and a QiaCube (QIAGEN), and cDNA was synthesized using the iScript cDNA Synthesis Kit (Bio-Rad) and a ProFlex thermocycler (Thermo Fisher Scientific). Target genes were amplified using PowerUp SYBR Green Master Mix and a QuantStudio 6 thermocycler (Thermo Fisher Scientific). Gene expression was calculated using the ΔΔCt method, with normalization to human *GAPDH* (sense: 5′-CTTCACCACCATGGAGAAGGC-3′, antisense: 5′-GGCATGGACTGTGGTCATGAG-3′) or mouse *Gapdh* (sense: 5′-TGCCCCCATGTTTGTGATG-3′, antisense: 5′- TGTGGTCATGAGCCCTTCC-3′). Target human gene primers were *PTCH1* (sense: 5′-GAAGAAGGTGCTAATGTCCTGAC − 3′, antisense: 5′-GTCCCAGACTGTAATTTCGCC -3′), *SMO* (5′-GAAGTGCCCTTGGTTCGGA − 3′, antisense: 5′-GCAGGGTAGCGATTCGAGTT − 3′) and *GLI1* (sense 5′-AGCCTTCAGCAATGCCAGTGAC-3′, antisense: 5′- GTCAGGACCATGCACTGTCTTG-3′). Target mouse gene primers were *Gli1* (sense 5′- GGTGCTGCCTATAGCCAGTGTCCTC-3′, antisense: 5′-GTGCCAATCCGGTGGAGTCAGACCC − 3′).

### Statistics

All experiments were performed with at least 3 biologic replicates and at least 3 technical replicates. Lines represent mean and error bars represent standard error of the mean. Local recurrence free and overall survival were estimated using the Kaplan-Meier method and compared by log-rank tests. ANOVA and two-tailed Student’s unpaired t-test were used to compare groups, and in all cases, statistical significance, as denoted by (*), was defined as *p* ≤ 0.05.

## Results and discussion

**Fig. 1:**
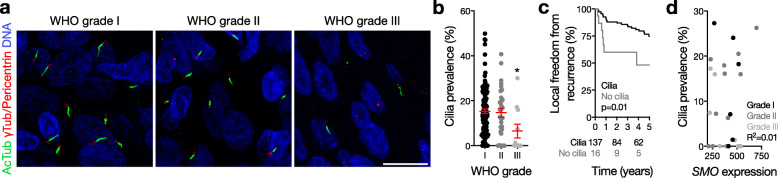
Meningiomas express primary cilia. **a** Confocal immunofluorescence microscopy for the ciliary marker acetylated tubulin (AcTub), the ciliary base/centriole markers gamma tubulin (γTub) and pericentrin, and DNA (DAPI) reveals that meningiomas express primary cilia. **b** Quantitative confocal immunofluorescence microscopy shows that WHO grade III meningiomas express less cilia than other meningiomas (*p = 0.04 ANOVA, *p* = 0.01 Student’s t test). **c** Consistent with loss of cilia in high grade meningiomas, local freedom from recurrence is shorter in meningiomas without primary cilia compared to meningiomas expressing primary cilia (log-rank test). **d** Nanostring transcriptional profiling demonstrates that *SMO* expression does not correlate with cilia prevalence in meningioma

To investigate mechanisms of Hedgehog signaling in meningioma, we created tissue microarrays comprised of 154 meningiomas from patients with comprehensive clinical follow-up data (median follow-up 4.3 years) who were treated with standard therapies at a single institution from 2003 to 2012 [14]. Tissue microarray tumors were re-reviewed according to current diagnostic criteria [13], revealing 113 WHO grade I (73%), 30 grade II (20%, atypical) and 11 grade III (7%, anaplastic) meningiomas in 105 women (68%) and 49 men (32%), as described in Supplementary Table S1. Using these microarrays, we performed immunofluorescence and confocal microscopy and discovered that meningiomas of all grades express primary cilia (Fig. 1a), but that cilia were less common in anaplastic meningiomas (Fig. 1b). Consequently, local freedom from recurrence was worse for meningiomas lacking primary cilia (Fig. 1c, Supplementary Table S1), a correlative observation likely attributable to meningioma grade that may not indicate a causative role for loss of cilia in meningioma tumorigenesis. To determine if cilia prevalence correlated with Hedgehog gene expression in meningiomas, we performed NanoString transcriptional profiling for *SMO* from 26 tumors, which failed to identify any associations between *SMO* expression and cilia prevalence in meningioma (Fig. 1d). Further, we identified no differences in overall survival according to ciliary length or prevalence; no differences in ciliary length or *SMO* expression according to meningioma grade; no differences in *SMO* expression according to ciliary length; no differences in local freedom from recurrence according to ciliary length or *SMO* expression; no differences in ciliary length or prevalence according to patient sex, prior treatment, or meningioma location; and no associations between ciliary length or prevalence and meningioma size or MIB1 labeling index (Supplementary Table S1). Thus, meningiomas can express the structure necessary for canonical Hedgehog signal transduction, but are less likely to do so when anaplastic (WHO grade III). In conjunction with the observation that Hedgehog pathway somatic variants are enriched in low grade meningiomas with favorable outcomes after treatment with standard therapies [19], our findings suggest that Hedgehog signaling does not underlie meningioma progression. In further support of this hypothesis, we show that cilia expression in meningioma does not correlate with expression of *SMO* in vivo. However, our data are limited by the fact that the genomic status of the meningiomas included in our tissue microarrays is unknown, and may not have included tumors with Hedgehog pathway variants, which are very rare in human meningiomas [19]. To shed light on this possible explanation, we identified 3 meningiomas harboring inactivating variants in *SUFU*, a negative regulator of the Hedgehog pathway, using clinical targeted next-generation sequencing [10]. Consistent with our hypothesis that ciliary expression does not correlate with Hedgehog signal transduction in meningioma, immunofluorescence and confocal microscopy revealed that all 3 meningiomas with loss of *SUFU* failed to express primary cilia (Supplementary Fig. S1). Of note, we have identified no meningiomas harboring *SMO* variants at our institution to date using either clinical targeted next-generation sequencing, or exploratory whole exome sequencing [20].

**Fig. 2:**
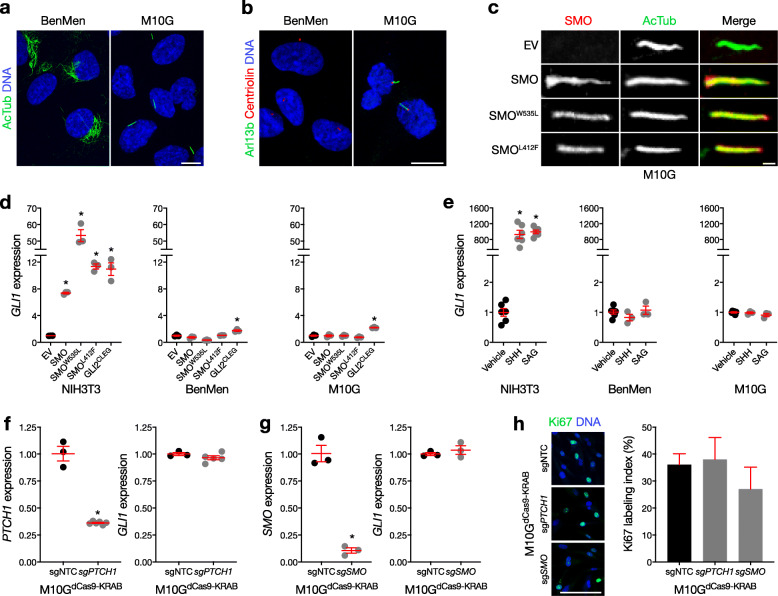
Primary meningioma cells accumulate Smoothened in cilia, but do not transduce ciliary Hedgehog signals. **a, b** Confocal immunofluorescence microscopy for the ciliary markers acetylated tubulin (AcTub) or Arl13b, the ciliary base/centriole marker Centriolin, and DNA (Hoechst) reveals that M10G meningioma cells express primary cilia, but BenMen meningioma cells do not, instead expressing AcTub in a perinuclear pattern. **c** Confocal immunofluorescence for SMO 72 h after transfection of *SMO*, oncogenic point substitutions of SMO (*SMO*^*W535L*^ or *SMO*^*L412F*^), or empty vector (EV) control, shows that exogenous SMO accumulates in the cilia of M10G meningioma cells. **d** qRT-PCR assessment of *GLI1* expression 72 h after transfection of *SMO*, *SMO*^*W535L*^*, SMO*^*L412F*^, constitutively active GLI2 (GLI2^CLEG^), or EV control, shows that over-expression of SMO activates the Hedgehog transcriptional program in NIH/3T3 cells, but not in BenMen or M10G meningioma cells. In contrast, over-expression of GLI2^CLEG^ mildly induces *GLI1* expression in meningioma cells (*p < 0.01 Student’s t test). **e** qRT-PCR assessment of *GLI1* expression 24 h after treatment with recombinant Sonic Hedgehog (SHH, 1 μg/ml), SMO agonist (SAG, 100 nM), or vehicle control, demonstrates that pharmacologic stimulation of the Hedgehog pathway activates the Hedgehog transcriptional program in NIH/3T3 cells, but not in BenMen or M10G meningioma cells (*p < 0.0001 Student’s t test). **f** qRT-PCR assessment of *GLI1* expression shows that CRISPRi suppression of *PTCH1* (sg*PTCH1*) compared to transduction of non-targeted sgRNAs (sgNTC) fails to activate the Hedgehog transcriptional program in M10G meningioma cells (*p < 0.0001 Student’s t test). **g** qRT-PCR assessment of *GLI1* expression shows that CRISPRi suppression of *SMO* (sg*SMO*) compared to transduction of sgNTC fails to suppress the Hedgehog transcriptional program in M10G meningioma cells (p < 0.0001 Student’s t test). **h** Quantitative confocal immunofluorescence microscopy for the proliferation marker Ki67 demonstrates that CRISPRi suppression of *PTCH1* or *SMO* compared to sgNTC fails to alter M10G meningioma cell proliferation. qRT-PCR data are normalized to *GAPDH* expression and EV, vehicle, or sgNTC controls as indicated. All experiments were performed at least 3 times with at least 3 technical replicates

To further interrogate Hedgehog signaling mechanisms in meningioma cells, we performed immunofluorescence and confocal microscopy for cilia in BenMen and M10G primary human meningioma cells, both of which were derived from WHO grade I tumors. We found that M10G cells expressed primary cilia, but BenMen cells did not (Fig. 2a, b). To determine if meningioma cells encode the Hedgehog transcriptional program, we expressed ciliary and post-ciliary activators of the Hedgehog pathway in primary meningioma cells, and assessed Smoothened accumulation in meningioma cilia, and quantified output of the Hedgehog transcriptional program. In parallel, we transfected BenMen and M10G meningioma cells with (i) wild type SMO; (ii) an oncogenic allele of SMO identified in BCC and medulloblastoma (SMO^W535L^) [18]; (iii) the most common SMO somatic variant identified in meningioma (SMO^L412F^) [2, 4, 5, 22], which is unresponsive to vismodegib in other cancers [20]; (iv) a constitutively active allele of GLI2 (GLI2^CLEG^), the primary activator of the Hedgehog transcriptional program downstream of cilia; or (v) empty vector (EV) control. We found that SMO, SMO^W535L^ and SMO^L412F^ all accumulated in cilia of M10G meningioma cells (Fig. 2c). However, when we assessed the Hedgehog transcriptional program in these cells using quantitative reverse transcriptase polymerase chain reaction (qRT-PCR) for the Hedgehog target gene *GLI1*, we found that over-expression of SMO, SMO^W535L^ and SMO^L412F^ failed to activate the Hedgehog transcriptional program in meningioma cells (Fig. 2d). In contrast, ciliated NIH3T3 cells, which encode the Hedgehog transcriptional program, dramatically induced *GLI1* expression in response to SMO, SMO^W535L^, SMO^L412F^ and GLI2^CLEG^ (Fig. 2d). Consistently, pharmacologic stimulation of the Hedgehog pathway induced *GLI1* expression from NIH3T3 cells, but not from meningioma cells (Fig. 2e). GLI2^CLEG^ mildly induced *GLI1* expression in meningioma cells (Fig. 2d), suggesting that somatic variants targeting the Hedgehog pathway downstream of cilia may activate Hedgehog signaling in meningioma. Nevertheless, there are currently no clinically tractable inhibitors of GLI transcription factors that could be used to treat meningioma patients.

To validate our finding that primary meningioma cells do not transduce ciliary Hedgehog signals, we generated ciliated M10G cells stably expressing the CRISPR interference (CRISPRi) components dCas9-KRAB [8], and stably suppressed *PTCH1* or *SMO* by transducing single guide RNAs (sgRNAs). qRT-PCR revealed that *PTCH1* suppression failed to activate the Hedgehog transcriptional program (Fig. 2f), and *SMO* suppression failed to inhibit the Hedgehog transcriptional program (Fig. 2g), compared to transduction of non-targeted sgRNA controls (sgNTC) in M10G^dCas9-KRAB^ cells. Moreover, immunofluorescence and quantitative confocal microscopy for the proliferation marker Ki67 demonstrated that neither sg*PTCH1* nor sg*SMO* altered the proliferation of M10G^dCas9-KRAB^ cells (Fig. 2h).

## Conclusions

In summary, our results reveal that meningiomas express primary cilia, but do not transduce ciliary Hedgehog signals. These data are consistent with larger studies of meningioma transcriptomics using RNA sequencing, which fail to identify aberrations in the Hedgehog pathway in these tumors [16, 20]. Thus, in the context of negative trials of Hedgehog pathway inhibitors for other cancers where the mechanisms of Hedgehog signaling were not fully understood [6], the data presented here suggest that further preclinical translation research is necessary before new clinical studies of Hedgehog pathway inhibitors are opened for meningioma patients.

## Supplementary information

**Additional file 1: Supplementary Fig. S1.** Meningiomas harboring inactivating variants in *SUFU*, a negative regulator of the Hedgehog pathway, do not express primary cilia. Confocal immunofluorescence microscopy for the ciliary marker acetylated tubulin (AcTub), the ciliary base/centriole markers Pericentrin and γTubulin (γTub), and DNA (DAPI) reveals that meningiomas harboring in activating variants in *SUFU* do not express primary cilia. Of note, 2 of 3 meningiomas harboring inactivating variants in *SUFU* also harbored inactivating variants in *NF2*.

**Additional file 2: Supplementary Table S1.** Meningioma characteristics. For binary variables such as skull base location, recurrent presentation, prior surgery, prior radiotherapy, GTR, local failure, and death, 1 denotes an event and 0 denotes the absence of an event. Male sex is denoted as 1, and female sex is denoted as 0. Radiotherapy target delineation software (MIM, Cleveland, OH) was used to contour and calculate meningioma volumes. Time to failure and survival time is shown in years. Ciliary length is shown in micrometers.

## Data Availability

Details about the methods used for ciliary immunofluorescence, NanoString transcriptional profiling, and molecular and cell biology experiments can be found in Methods. All raw data are available in Supplementary Table S1.

## Abbreviations

GLI: Glioma associated oncogene
GTR: Gross total resection
LI: Labeling index
MIB1: Mindbomb E3 Ubiquitin Protein Ligase 1
NIH3T3: National Institutes of Health mouse fibroblast 3T3 cells
OTHR: Other
PD: Progressive disease
PTCH: Patched
RT: Radiotherapy
SMO: Smoothened
TX: Treatment
UNKN: Unknown
