# Supplementary material for: Meningioma cells express primary cilia but do not transduce ciliary Hedgehog signals
Source: Acta Neuropathol Commun. 2020 Jul 20;8:114. doi: 10.1186/s40478-020-00994-7 (PMC7370519; doi:10.1186/s40478-020-00994-7)

Supplementary Fig. S1 Meningiomas harboring inactivating variants in *SUFU*, a negative regulator of the Hedgehog pathway, do not express primary cilia.

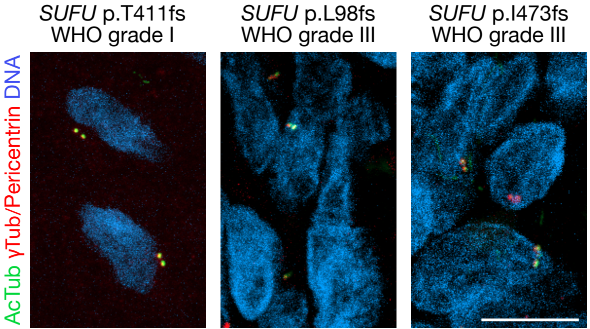

Supplement: Supplementary file 1 — Additional file 1: Supplementary Fig. S1. Meningiomas harboring inactivating variants in SUFU, a negative regulator of the Hedgehog pathway, do not express primary cilia. Confocal immunofluorescence microscopy for the ciliary marker acetylated tubulin (AcTub), the ciliary base/centriole markers Pericentrin and γTubulin (γTub), and DNA (DAPI) reveals that meningiomas harboring in activating variants in SUFU do not express primary cilia. Of note, 2 of 3 meningiomas harboring inactivating variants in SUFU also harbored inactivating variants in NF2. [file 40478_2020_994_MOESM1_ESM.pdf]
